# Motivating Time-Inconsistent Agents: A Computational Approach

**DOI:** 10.1007/s00224-018-9883-0

**Published:** 2018-08-07

**Authors:** Susanne Albers, Dennis Kraft

**Affiliations:** Department of Informatics, Technical University of Munich, Boltzmannstr. 3, 85748 Garching, Germany

**Keywords:** Approximation algorithms, Behavioral economics, Commitment devices, Computational complexity, Time-inconsistent preferences

## Abstract

We study the complexity of motivating time-inconsistent agents to complete long term projects in a graph-based planning model proposed by Kleinberg and Oren (2014). Given a task graph *G* with *n* nodes, our objective is to guide an agent towards a target node *t* under certain budget constraints. The crux is that the agent may change its strategy over time due to its present-bias. We consider two strategies to guide the agent. First, a single reward is placed at *t* and arbitrary edges can be removed from *G*. Secondly, rewards can be placed at arbitrary nodes of *G* but no edges must be deleted. In both cases we show that it is NP-complete to decide if a given budget is sufficient to keep the agent motivated. For the first setting, we give complementing upper and lower bounds on the approximability of the minimum required budget. In particular, we devise a $(1+\sqrt {n})$-approximation algorithm and prove NP-hardness for ratios greater than $\sqrt {n}/3$. We also argue that the second setting does not permit any efficient approximation unless P = NP.

## Introduction

In behavioral economics people’s tendency to change long term plans for no apparent reason is known as *time-inconsistent behavior*. Examples of such behavior are plenty in every day life, including academia. Consider, for instance, a referee who agrees to evaluate a scientific proposal. Despite good intentions, the referee gets distracted and never submits a report. Or consider a student who enrolls in a course. After completing the first homework assignments, the student drops out without earning any credit. In general, these situations have a reoccurring pattern: An agent makes a plan to complete a set of tasks in the future, but changes the plan at a later point in time. This behavior is sometimes the result of unforeseen circumstances. However, in many cases the plan is changed or abandoned even if the circumstances stay the same. This paradox behavior of *procrastination* and *abandonment* might severely affect the performance of agents in an economic or social domain, see e.g. [1, 12, 14].

A sensible explanation for time-inconsistent behavior is that agents assign disproportionately greater value to the present than to the future. For example, consider a simple *car wash problem* in which Alice commissions Bob to wash her car. Each day Bob can either do the chore or postpone it to the next day. However, the longer he waits, the dirtier the car gets. On day *i* cleaning the car incurs a cost of *i*/50 while the cost of waiting another day is 0. After completing the task, Bob will receive a reward of 1 from Alice. Because Bob is present-biased, he perceives any current cost according to its true value, but discounts future costs and rewards by a factor of *β* ∈ [0, 1]. On day *i* he compares the cost of washing the car right away, which is *i*/50, to his perceived cost of washing it on the next day, which is *β*(*i* + 1)/50. Suppose that *β* = 1/3. Since *i*/50 > *β*(*i* + 1)/50, he procrastinates with good intentions of doing the job on the following day. On day *i* = 50, Bob’s perceived cost for washing the car on the next day or any of the following days is at least *β*(50 + 1)/50. This exceeds his perceived reward of *β* and therefore he abandons the project.

### Related Work

There exists an extensive body of work on time-inconsistent behavior, see again [1, 12, 14]. A common idea thorough the literature is that time-inconsistent behavior arises because agents discount the value and cost of future events disproportionately. Remember the car wash problem. In this scenario we have considered a special case of *quasi-hyperbolic discounting*, which is a well established discounting model introduced by [10]. We build on work by [7], who proposed a graph-based model to capture the behavior of quasi-hyperbolic discounting agents in general planning problems. Since its introduction, their work sparked an active line of research at the intersection of economics, mathematics and computer science, see e.g. [2, 3, 6, 8, 9].

We give a formal definition of our model in Section 2. Essentially, it consists of a directed acyclic task graph *G* with *n* nodes. Each node represents a certain state of the project, whereas the edges are tasks necessary to transition between states. The workload of individual tasks is modeled by edge costs. To complete the project, an agent with bias factor *β* ∈ [0, 1] must move from a designated source *s* to a target *t*. As a motivation, rewards are placed on the nodes of *G*. When located at some node of *G*, the agent considers all possible paths to *t*. However, because of its time inconsistency, the agent only evaluates the cost of incident edges accurately. All other costs and rewards are discounted by a factor of *β*. Let *P* be a path that minimizes the agent’s *perceived net cost*. If this cost is at most 0, the agent traverses the first edge of *P* and then reassesses its plan. Otherwise the agent abandons the project. A graph in which the agent always reaches *t* is called *motivating*.

We take the perspective of a project designer, whose main objective is budget-efficiency. In other words, we try to minimize the reward we must spend to get the project completed. In general, various strategic arrangements can be made to increase budget-efficiency. For instance, in the car wash problem Alice can introduce a deadline to keep Bob from procrastinating. As we show in Section 2, this is beneficial to both of them. Because the aim of such an arrangement is to commit the agent to finish the project, it is often called a *commitment device*, see e.g. [4]. Note that deadlines belong to a broader range of popular commitment devices that reduce the agent’s set of choices [12, 13]. In the graph-based model we can implement such devices by removing the corresponding edges from *G* [7].

Another common commitment device is the placement of rewards at intermediate states of the project, see e.g. [13]. In the graph-based model, this can be achieved by placing rewards at non-terminal nodes of *G*. We call such an assignment a *reward configuration*. This approach is especially interesting if the project designer’s budget is only affected by rewards that are actually collected by the agent. As we show in Section 2, this allows the construction of *exploitative* projects in which the agent is motivated by rewards it never claims. Kleinberg and Oren [7] pose the complexity of computing motivating subgraphs and reward configurations as two important open problems.

In recent work, [15] address both problems. First, they show that it is NP-hard to decide if *G* contains a motivating subgraph for a fixed reward at *t*. Secondly, they give an NP-hardness result and a 2*n*-approximation algorithm for three variations of the reward configuration problem: One with positive rewards, one with arbitrary rewards and one in which all rewards placed on *G* must be collected. In each setting, the project designer pays the absolute sum laid out.

### Our Contribution

We thoroughly analyze the complexity and approximability of computing motivating subgraphs and reward configurations. In Section 3, we settle the complexity of finding a motivating subgraph for a fixed reward at *t*. First, we show that the problem is polynomially solvable if *β* = 0 or *β* = 1. We then prove that it is NP-complete to decide the existence of a motivating subgraph for general *β* ∈ (0, 1). Tang et al. [15] showed NP-hardness via a reduction from 3-SAT. In contrast, we use reduction from *k* DISJOINT CONNECTING PATHS. We believe this reduction to be simpler. More importantly, we are able to generalize the reduction to obtain a hardness of approximation result at a later point.

Considering the hardness of the motivating subgraph problem, Section 4 focus on an optimization version of the problem. More formally, we want to compute the minimum reward that must be placed at *t* such that *G* contains a motivating subgraph. We propose a simple $(1+\sqrt {n})$-approximation algorithm that outputs the reward and a corresponding motivating subgraph. As our main technical contribution, we show that this approximation is asymptotically tight. In particular, we prove that the problem cannot be approximated efficiently within a ratio less than $\sqrt {n}/3$ unless P = NP. Thus, we resolve the approximability of the motivating subgraph problem up to a small constant factor.

Finally, Section 5 explores the problem of finding reward configurations within a fixed total budget of at most *b*. We examine a version of the problem that, in our view, is the most interesting one. First, only positive rewards may be laid out. This assumption avoids the issue of finding a sensible way to account for negative rewards in the designer’s budget. A version with negative rewards that do not affect the budget can be found in [2]. Secondly, the designer must only pay for rewards that are actually collected by the agent. This setting is fundamentally different from the settings analyzed by Tang et al. as it allows exploitative solutions. We show that the problem can be solved in polynomial-time if *β* = 0 or *β* = 1. Using a reduction from SET PACKING, we prove that deciding the existence of a motivating reward configuration is NP-complete for general *β* ∈ (0, 1), even if *b* = 0. This immediately implies that the optimization problem of finding the minimum *b* for which a motivating reward configuration exists cannot be approximated efficiently within any ratio greater or equal to 1 unless P = NP.

## The Graph-Based Model

In the following, we present [7] model. Let *G* = (*V*,*E*) be a finite directed acyclic graph. Associated with each edge (*v*,*w*) is a non-negative cost *c*_*G*_(*v*,*w*). Furthermore, the project designer may lay out positive rewards *r*_*G*_(*v*) at arbitrary nodes *v*. We call *r* a *reward configuration*. An agent with bias factor *β* ∈ [0, 1] has to incrementally construct a path from a source *s* to a target *t*. Located at some node *v* different from *t*, the agent evaluates its *lowest perceived net cost*. For this purpose it considers all paths *P* from *v* to *t*. However, only the initial edge of *P* is accounted for by its actual value. All other costs and rewards along *P* are discounted by a factor of *β*. More precisely, let *d*_*G*,*r*_(*w*) denote the cost of a cheapest path from some node *w* to *t* with respect to the actual costs and rewards. Note that although *d*_*G*,*r*_(*w*) might be negative depending on *r*, no negative cycles can occur as *G* is acyclic. If no path exists, we assume that *d*_*G*,*r*_(*w*) = *∞*. The lowest perceived net cost is defined as *ζ*_*G*,*r*_(*v*) = min{*c*_*G*_(*v*,*w*) + *βd*_*G*_(*w*)∣(*v*,*w*) ∈ *E*} if *v* has at least one outgoing edge. Otherwise, *ζ*_*G*,*r*_(*v*) = *∞*. If *ζ*_*G*,*r*_(*v*) > 0, then the agent has no motivation to continue the project and abandons. Conversely, if *ζ*_*G*,*r*_(*v*) ≤ 0, the agent traverses an edge (*v*,*w*) for which *c*_*G*,*r*_(*v*,*w*) + *βd*_*G*,*r*_(*w*) = *ζ*_*G*,*r*_(*v*). Ties are broken arbitrarily. Note that the agent could take more than one path from *s* to *t*. A project is called motivating if the agent successfully reaches *t* along all such paths. To simplify our notation, we omit *G* and *r* in the index of *c*, *r*, *d* and *ζ* whenever the graph and reward configuration is clear from context.

**Fig. 1:**
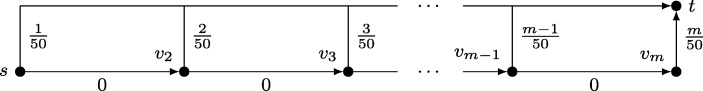
Task graph of the car wash problem

To illustrate the model, we consider the car wash problem from Section 1 once more. Assume that Alice’s car must be washed during the next *m* days with *m* > 50. The task graph *G* is depicted in Fig. 1. For each day *i* with 1 ≤ *i* ≤ *m* there is a node *v*_*i*_. Let *v*_1_ be the source. There is an edge (*v*_*i*_, *t*) of cost *i*/50 that represents the task of washing the car on day *i*. To keep the drawing simple, the edges (*v*_*i*_, *t*) merge in Fig. 1. Furthermore, for every *i* < *m* there is an edge (*v*_*i*_, *v*_*i*+ 1_) of cost 0 to postpone the task to the next day. Assume for now that Bob is located at some *v*_*i*_ with *i* < *m*. His perceived cost for procrastinating is at least *β*(*i* + 1)/50. This bound is tight if he plans to traverse (*v*_*i*_, *v*_*i*+ 1_) and then (*v*_*i*+ 1_, *t*). Alternatively, his perceived cost for using (*v*_*i*_, *t*) and washing the car on day *i* is *i*/50. Recall that Alice offers Bob a single reward *r*(*t*) = 1 upon completing the car wash. Furthermore, *β* = 1/3. As a result, the minimum perceived net cost is *ζ*(*v*_*i*_) = *β*(*i* + 1)/50 − *β*. We conclude that Bob always prefers to wash the car on the next day instead of doing it right away. Moreover, for *i* < 50 it holds true that *ζ*(*v*_*i*_) ≤ 0. This means that during the first 49 days, Bob moves along (*v*_*i*_, *i* + 1) believing that he will finish the project the next day. However, once Bob reaches *v*_50_ he suddenly realizes that *ζ*(*v*_50_) > 0 and abandons. Therefore, the car wash problem in its current form is not motivating.

Next, assume that (*v*_16_, *v*_17_) is deleted from *G*. This can be thought of as a deadline set by Alice at day *i* = 16. Let *G*^′^ be the resulting subgraph. When Bob reaches *v*_16_, he cannot procrastinate anymore but must wash the car to get a reward. The perceived net cost is $\zeta _{G^{\prime }}(v_{16}) = 16/50 - \beta = -1/75$. Because this is less than 0, he washes the car. This makes *G*^′^ a motivating subgraph. It is interesting to note that no reward configuration in *G* is motivating for a budget of *b* < (*m*/50)/*β*. The reason is that no matter how rewards are placed, Bob always prefers to procrastinate until the very last day.

To illustrate the power of reward configurations, we consider a second scenario. Suppose that Alice offers Bob a new deal. If he first washes her car, which by now incurs a cost of 1, and afterwards also mows her lawn, which has a cost of 6, he receives a reward of 10. What Bob is unaware of is that Alice does not care about the lawn. Instead, she tries to get Bob to wash the car for free. We model this project with a task graph *G* consisting of a path from *s* to *t* via the intermediate node *v* and another path from *v* to *t* via *w*. The edge (*s*,*v*) corresponds to the car wash and has a cost of 1. Furthermore, (*v*,*w*) corresponds to mowing of the lawn and has a cost of 6. The edges (*v*,*t*) and (*w*,*t*) are of cost 0. Assuming that *β* = 1/3, there is a reward configuration *r* for which Bob will wash the car but will not claim a reward. Suppose Alice sets *r*(*w*) = 10. In this case, Bob traverses (*s*,*v*) with a minimum perceived net cost of *ζ*(*s*) = − 1/3 along the path *s*,*v*,*w*,*t*. When at *v*, Bob reevaluates the net cost for traversing (*v*,*w*) to 8/3. In contrast, finishing the project right away along (*v*,*t*) has cost 0. As a result, Bob changes his plan and moves to *t* without collecting any reward.

## Finding Motivating Subgraphs

In this section, we assume that the project designer may only place a single reward at *t*. This way, no exploitative reward configurations are possible. We first argue that the problem of finding a motivating subgraph can be solved in polynomial-time if *β* = 0 or *β* = 1. Although this claim might seem intuitive, we are able to generalize the idea to show the existence of an $(1+\sqrt {n})$-approximation algorithm for general *β* ∈ (0, 1) in Section 4.

### **Proposition 1**

*Ifβ* = 0 *orβ* = 1*,it is possible to find a motivating subgraph in polynomial-time for arbitraryr*(*t*) ≥ 0*.*

### Proof

First, assume *β* = 0. Because the agent has no value for future rewards, it must walk along a path of cost 0. Otherwise, it would abandon once it encounters an edge of positive cost. If such a path exists, it itself is a motivating subgraph. Conversely, if no such path exists, no subgraph can be motivating. Next, assume *β* = 1. In this case, the agent behaves time-consistent and follows a cheapest path from *s* to *t*. Therefore, *G* contains a motivating subgraph if and only if there is a path from *s* to *t* with a total edge cost less or equal to *r*(*t*). Any subgraph containing such a path is motivating. Clearly, if a motivating subgraph exists, it can be found efficiently in both scenarios, i.e. *β* = 0 and *β* = 1.

Unfortunately, computing motivating subgraphs for general *β* ∈ (0, 1) is more challenging. We give evidence for this in Theorem 1 by showing that the corresponding decision problem, which we name MOTIVATING SUBGRAPH (MS), is NP-complete for general *β* ∈ (0, 1).

### **Definition 1** (MS)

Given a task graph *G*, a reward *r*(*t*) ≥ 0 and a bias factor *β* ∈ [0, 1], decide the existence of a motivating subgraph of *G*.

To prove NP-completeness of MS, we must first show that MS is contained in NP. For this purpose we argue that it can be decided in polynomial-time whether a task graph is motivating for a given reward configuration. Note that a naive approach that simply simulates the agents walk through *G* must fail as the agent might take more than one path whenever it is indifferent between two options. A possible solution that preserves polynomial-time bounds is presented in the following proposition.

### **Proposition 2**

*For any task graphG,reward configurationrand bias factorβ* ∈ [0, 1]*,it can be decided in polynomial-time ifGis motivating.*

### Proof

We modify *G* in the following way. For each node *v* we calculate the lowest perceived net cost *ζ*_*G*,*r*_(*v*). Next, we take a copy of *G*, say *G*^′^, in which we remove all edges (*v*,*w*) for which *ζ*_*G*,*r*_(*v*) < *c*_*G*_(*v*,*w*) + *βd*_*G*,*r*_(*w*) or *ζ*_*G*,*r*_(*v*) > 0. In other words, we remove all edges from *G*^′^ that do not minimize the agent’s perceived net cost or are not motivating. Let *V*^′^ be the set of all nodes that can be reached from *s* in *G*^′^. Observe that *V*^′^ contains exactly those nodes that might be visited by the agent in *G*. Clearly, *G* is motivating if and only if the agent can reach *t* from all nodes of *V*^′^ via some path in *G*^′^ . This condition can be checked in polynomial-time.

To show NP-hardness, we use a reduction from *k* DISJOINT CONNECTING PATHS (*k*-DCP), which is defined as follows [5]:

### **Definition 2** (*k*-DCP)

Given a graph *H* containing *k* disjoint pairs of nodes (*s*_1_, *t*_1_),…,(*s*_*k*_, *t*_*k*_), decide if *H* has *k* mutually node-disjoint paths, one connecting every *s*_*i*_ to the corresponding *t*_*i*_.

Lynch [11] showed that *k*-DCP is NP-complete if *H* is undirected. A simple modification of Lynch’s reduction, which can be found in the appendix, implies that *k*-DCP is also NP-complete if *H* is directed and acyclic.

Before we tackle Theorem 1, we want to draw attention to a useful price structure that is common to all our reductions presented in this work. Imagine a directed path along *k* + 1 edges, such that the *i*-th edge has a cost of (1 − *β*)^*k*+ 1−*i*^. According to the following Lemma, the agent’s perceived cost for following the path to its end is 1 at every node except for the last.

### **Lemma 1**

*For every positive integerkand bias factorβ* ∈ [0, 1] *it holds truethat*

$$(1 - \beta)^{k} + \beta\left( \sum\limits_{i = 0}^{k-1}(1 - \beta)^{i}\right) = 1. $$

The correctness of Lemma 1 follows from basic calculus, in particular from the geometric series. A formal proof can be found in the appendix. We are now ready to show NP-completeness of MS.

### **Theorem 1**

*MS is NP-complete for any bias factorβ* ∈ (0, 1)*.*

### Proof

By Proposition 2, any motivating subgraph *G*^′^ serves as a certificate for a “yes”-instance of MS. Consequently, MS is in NP. To complete the proof, we establish NP-hardness via a polynomial reduction from *k*-DCP.

**Fig. 2:**
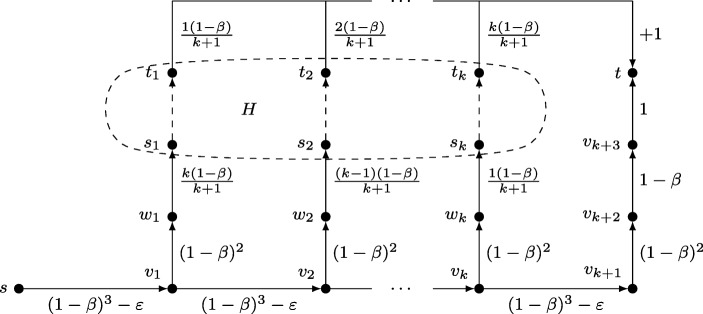
Reduction from a general *k*-DCP instance: *H*

Consider an instance ${\mathcal I}$ of *k*-DCP consisting of a directed acyclic graph *H* and *k* disjoint node pairs (*s*_1_, *t*_1_),…,(*s*_*k*_, *t*_*k*_). Our goal is to embed *H* into the task graph *G* of an MS instance ${\mathcal J}$ such that *G* has a motivating subgraph if and only if *H* has *k* disjoint connecting paths. For this purpose we assume that the encoding length of *β* ∈ (0, 1) is polynomial in that of ${\mathcal I}$ and set *r*(*t*) = 1/*β* . The task graph *G*, which is illustrated in Fig. 2, is constructed as follows:

To get from *s* to *t*, the agent must follow the so called main path along intermediate nodes *v*_1_,…,*v*_*k*+ 3_. The first *k* + 1 edges of this main path each have a cost of (1 − *β*)^3^ − *ε*, with *ε* being a positive constant satisfying

$$\varepsilon < \min\left\{\beta \frac{1-\beta}{k + 1}, \beta\frac{(1-\beta)^{3}}{1+\beta}\right\}. $$

The last three edges have a cost of (1 − *β*)^2^, 1 − *β* and 1, respectively. To keep the agent motivated, we introduce *kshortcuts* that connect every *v*_*i*_ with 1 ≤ *i* ≤ *k* to *t* via the embedding of *H*. More formally, the *i*-th shortcut starts at *v*_*i*_ and is routed through a distinct node *w*_*i*_ via an edge of cost (1 − *β*)^2^. Node *w*_*i*_ is then connected to *s*_*i*_ via an edge of cost (*k* + 1 − *i*)(1 − *β*)/(*k* + 1). Finally, *t*_*i*_ is connected to *t* via an edge of cost *i*(1 − *β*)/(*k* + 1) + 1. To keep Fig. 2 simple, the edges (*t*_*i*_, *t*) are merged and their cost is depicted as two terms, namely *i*(1 − *β*)/(*k* + 1) and + 1. Note that the prices of (*w*_*i*_, *s*_*i*_) and (*t*_*i*_, *t*) complement each other, i.e. they sum to (1 − *β*) + 1. The edges of *H* all have a cost of 0. The resulting graph *G* is acyclic and its encoding length polynomial in ${\mathcal I}$. It remains to show, that ${\mathcal J}$ has a solution if and only if ${\mathcal I}$ has one.

(⇒) First, suppose ${\mathcal I}$ has a solution, i.e. there exist *k* node-disjoint connecting paths. Let *G*^′^ be a subgraph of *G* obtained by deleting all edges of *H* that are not part of one of these paths. Furthermore, let *s* = *v*_0_ and assume the agent is located at *v*_*i*_ with 0 ≤ *i* ≤ *k*. According to Lemma 1, the agent perceives a net cost of − *ε* for taking the (*i* + 1)-st shortcut or if *i* = *k* for following the main path. In contrast, if 0 < *i* ≤ *k*, the perceived net cost of the *i*-th shortcut is 0. As a result, the agent follows the main path to *v*_*k*+ 1_ and then for lack of other options continues to *t*. We conclude that *G*^′^ is a motivating subgraph of *G*.

(⇐) To prove the other direction, assume that there are no *k* node-disjoint paths in *H* and let *G*^′^ be an arbitrary subgraph of *G*. Our goal is to argue that *G*^′^ cannot be motivating.

It is crucial to observe that *G*^′^ is only motivating if the agent never leaves the main path. Otherwise, it would have to pass some node *t*_*i*_ on its way to *t*. At this point the agent perceives a net cost of *i*(1 − *β*)/(*k* + 1) + 1 − 1 > 0 and abandons. We therefore focus on subgraphs *G*^′^ in which the agent stays on the main path. Of course, any such *G*^′^ must contain the main path. We say the *i*-th shortcut of *G*^′^ is *degenerate* if the cost of a cheapest path from *v*_*i*_ to *t* via *w*_*i*_ is different from the *target value𝜗* = (1 − *β*)^2^ + (1 − *β*) + 1. In particular, the *i*-th shortcut is degenerate if there is no path from *v*_*i*_ to *t* via *w*_*i*_ in which case the cost is infinite. Note that by construction, every degenerate shortcut must miss the target value by (1 − *β*)/(*k* + 1) or more.

We first argue that there is at least one degenerate shortcut in *G*^′^. For the sake of contradiction assume that no such shortcut exists. This means that there is a cheapest path *P*_*i*_ from *v*_*i*_ to *t* via *w*_*i*_ for all 1 ≤ *i* ≤ *k*. By construction, *P*_*i*_ must contain (*w*_*i*_, *s*_*i*_). Because total cost of *P*_*i*_ should sum up to *𝜗*, it follows that *P*_*i*_ must end in (*t*_*i*_, *t*). Furthermore, *P*_*i*_ must be node-disjoint from all other paths *P*_*j*_ with *j* < *i*. Otherwise, *P*_*i*_ would not be a shortest path from *v*_*i*_ to *t* considering that the cost of (*t*_*j*_, *t*) is less than the cost of (*t*_*i*_, *t*). As a result, the subpaths of *P*_*i*_ between *s*_*i*_ and *t*_*i*_ correspond to *k* node-disjoint paths in *H*. This contradicts the assumption that ${\mathcal I}$ has no solution.

Now that we have established the existence of a degenerate shortcut in *G*^′^, we distinguish two cases: Either there exists a degenerate shortcut *i* such that the cost of a cheapest path from *v*_*i*_ to *t* via *w*_*i*_ is less than *𝜗*, or every degenerate shortcut costs more than *𝜗*. We start with the first case. Let *i* be the largest index of a degenerate shortcut such that the cheapest path from *v*_*i*_ to *t* via *w*_*i*_ is less than *𝜗*. When located at *v*_*i*_, the agent’s perceived net cost along (*v*_*i*_, *w*_*i*_) is less or equal to

$$(1-\beta)^{2} + \beta\left( (1-\beta)+ 1-\frac{1-\beta}{k + 1}\right) - 1 = \beta\frac{1-\beta}{k + 1}<-\varepsilon. $$

The equality can be seen easily by means of Lemma 1. Furthermore, the inequality holds by choice of *ε*. The agent’s second option is to stay on the main path. However, the fact that all subsequent shortcuts cost *𝜗* or more implies that the agent’s perceived net cost along (*v*_*i*_, *v*_*i*+ 1_) is at least − *ε*. Clearly, this is a contradiction to the assumption that the agent stays on the main path. We conclude that *G*^′^ is not motivating.

We now take a look at the second case. Suppose that the *i*-th shortcut is degenerate and assume that the agent plans to travel from *v*_*i*− 1_ to *t* along the main path. Also recall that *s* = *v*_0_. The agent has two options. If it plans to follow the *i*-th shortcut, its perceived net cost is greater or equal to

$$(1-\beta)^{3} -\varepsilon + \beta\left( (1-\beta)^{2}+(1-\beta) + 1 + \frac{1-\beta}{k + 1}\right) - 1 = \beta \frac{1-\beta}{k + 1}-\varepsilon > 0. $$

Again, the inequality holds by choice of *ε*. If the agent plans to go one edge further along the main path instead, i.e. traversing (*v*_*i*_, *v*_*i*+ 1_) and possibly taking a shortcut of index *j* with *j* > *i*, its perceived net cost is at least

$$(1-\beta)^{3} -\varepsilon + \beta((1-\beta)^{3} -\varepsilon + (1-\beta)^{2}+(1-\beta) + 1) - 1 \!=\! (1+\beta)\left( \beta\frac{(1-\beta)^{3}}{1+\beta} -\varepsilon\right)\!>\!0. $$

This holds true because no shortcut is of cost less than *𝜗*. In both cases the perceived net cost is greater than 0 by choice of *ε*. Consequently, the agent is not motivated to follow the main path at *v*_*i*− 1_. As argued before, this means that *G*^′^ cannot be motivating and completes the proof.

## Approximating Optimal Subgraphs

Considering that the decision problem MS is NP-hard, the next and arguably natural question is whether good approximation algorithms exist. Therefore, we restate MS as an optimization problem that we call MS-OPT.

### **Definition 3** (MS-OPT)

Given a task graph *G* and a bias factor *β* ∈ (0, 1), determine the minimum reward *r*(*t*) such that *G* contains a motivating subgraph.

We present two simple approximation algorithms: one that performs well for small values of *β* and one that leads to good solutions for large *β*. The algorithms return a reward *r*(*t*) as well as a corresponding motivating subgraph *G*^′^. Combining both algorithms eventually yields a general approximation algorithm with a ratio of $(1+\sqrt {n})$ for any *β* ∈ (0, 1).

First, we assume that *β* is small. Because the agent is highly oblivious to the future, it is sensible to guide it along a path with minimal maximum edge cost. Paths with this property are called *minmax paths*. A minmax path can be computed easily in polynomial-time. For instance, starting with an empty subgraph, the edges of *G* can be inserted in non-decreasing order of cost until *s* and *t* become connected for the first time. Any path from *s* to *t* in the resulting subgraph is a minmax path. Our first algorithm, called MinmaxPathApprox, computes a minmax path *P* from *s* to *t* and returns a subgraph *G*^′^ whose edges are that of *P*. Furthermore, the reward *r*(*t*) is chosen in such a way that $\max \{\zeta _{G^{\prime },r}(v) \mid v \in P\} = 0$. Clearly, this is sufficient to make *G*^′^ motivating.

### **Proposition 3**

MinmaxPathApprox*has an approximation ratio of*1 + *βn.*

### Proof

Let *c* denote the maximum cost among the edges of the minmax path *P* computed by MinmaxPathApprox. By definition of *P*, the agent must encounter an edge of cost *c* or more in any subgraph that connects *s* with *t*. Thus the optimal reward is lower bounded by *c*/*β*. Conversely, the cost of every edge in *P*, of which there are at most *n* − 1, is *c* or less. This means that the reward returned by MinmaxPathApprox is upper bounded by *r*(*t*) ≤ *c*/*β* + (*n* − 2)*c* ≤ *c*/*β* + *nc*. From this the desired approximation ratio of 1 + *βn* follows immediately.

Next, suppose that *β* is large and the agent is hardly present-biased at all. Our second algorithm, called CheapestPathApprox, simply computes a path *P* of minimum cost from *s* to *t* and returns a subgraph *G*^′^ containing the edges of *P*. Again, the algorithm chooses *r*(*t*) in such a way that $\max \{\zeta _{G^{\prime },r}(v) \mid v \in P\} = 0$.

### **Proposition 4**

CheapestPathApprox*has an approximation ratio of*1/*β.*

### Proof

Let *P* be the path computed by CheapestPathApprox and *c* the total cost of *P*. At any node *v* of *P* the agent’s perceived net cost is at most $d_{G^{\prime },r}(v) - \beta r(t)$, which is less than *c* − *βr*(*t*). The reward returned by CheapestPathApprox is therefore at most *c*/*β*. Conversely, when located at *s*, the agent perceives a cost of at least *βc* in any subgraph of *G*, including the optimal one. Consequently, a reward of at least *c* is need to motivate the agent. This establishes the approximation ratio of 1/*β*.

It is interesting to see how MinmaxPathApprox and CheapestPathApprox generalize the algorithmic ideas of Proposition 1. If we combine the two and use MinmaxPathApprox whenever $\beta \leq 1/\sqrt {n}$ and CheapestPathApprox otherwise, we obtain a general approximation algorithm called CombinedApprox. Propositions 3 and 4 directly imply the following result.

### **Theorem 2**

CombinedApprox *has an approximation ratio* *of* $1+\sqrt {n}$ *.*

Although the algorithmic techniques of CombinedApprox are simple, Theorem 3 implies that up to a small constant factor the approximation ratio is the best we can hope for in polynomial-time. To prove the theorem, the following inequality will come in handy.

### **Lemma 2**

*For any integerϱwithϱ* ≥ 1 *it holds truethat*

$$\left( 1-\frac{1}{3\varrho+ 3}\right)^{3\varrho+ 3} > \frac{1}{3}. $$

Similar to Lemma 1, verifying Lemma 2 only requires basic calculus. Refer to the appendix for a proof. We are mow ready to establish Theorem 3.

### **Theorem 3**

*MS-OPT is NP-hard to approximate within a ratio less than* $\sqrt {n}/3$ *.*

### Proof

To show hardness of approximation, we use another reduction from *k*-DCP. Let ${\mathcal I}$ be a *k*-DCP instance consisting of a directed acyclic graph *H* and *k* disjoint node pairs (*s*_1_, *t*_1_),…,(*s*_*k*_, *t*_*k*_). Furthermore, let *ϱ* be an arbitrary positive integer. The best choice of *ϱ* will be determined later. Our goal is to construct an instance ${\mathcal J}$ of MS-OPT that consists of a task graph *G* and has the following two properties: (a) If ${\mathcal I}$ has a solution, then *G* has a subgraph that is motivating for a reward of *r*(*t*) = 1/*β*. (b) If ${\mathcal I}$ does not have a solution, then no subgraph of *G* is motivating for a reward of *r*(*t*) = *ϱ*/*β* or less. Consequently, any algorithm achieving an approximation ratio of *ϱ* or better must solve ${\mathcal I}$.

Unlike Theorem 1, the bias factor cannot be chosen arbitrarily anymore. Considering that Proposition 4 gives a (1/*β*)-approximation, *β* must be less than 1/*ϱ*. For convenience, we set *β* = 1/(3*ϱ* + 3). From a structural point of view, the task graph *G* consists of two units: the embedding unit and the amplification unit. The first unit contains an embedding of *H*, while the second unit amplifies approximation errors occurring in the embedding unit.

**Fig. 3:**
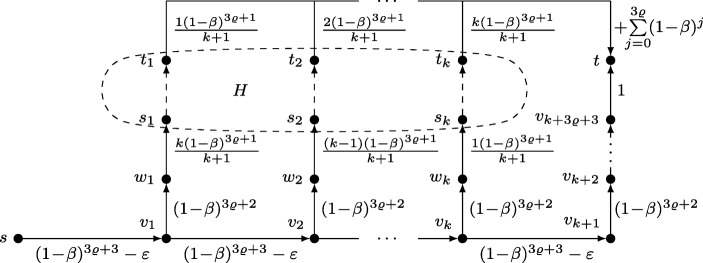
Embedding unit

The overall structure of the embedding unit, which is depicted in Fig. 3, is similar to the graph of Theorem 1. There exists a *main path* and *kshortcuts* that link to the embedding of *H*. However, there are some differences. First, the main path starts at the last node of the amplification unit $u_{9\varrho ^{2}}$ and passes *k* + 3*ϱ* + 3 intermediate nodes *v*_1_,…,*v*_*k*+ 3*ϱ*+ 3_ before it ends in *t*. The first *k* + 1 edges of the main path each have a cost of (1 − *β*)^3*ϱ*+ 3^ − *ε*, where *ε* is a positive value satisfying

$$\varepsilon < \min\left\{\beta\frac{(1-\beta)^{3\varrho+ 1}}{k + 1}, \beta\frac{(1-\beta)^{3\varrho+ 3}}{1+\beta}, \frac{1}{1+\varrho}, (1-\beta)^{3\varrho+ 3}-\frac{1}{3}\right\}. $$

Note that (1 − *β*)^3*ϱ*+ 3^ − 1/3 is positive according to Lemma 2. The remaining edges (*v*_*i*_, *v*_*i*+ 1_) of the main path, with *k* < *i* ≤ *k* + 3*ϱ* + 3 and *t* = *v*_*k*+ 3*ϱ*+ 3 + 1_, have an increasing cost of (1 − *β*)^*k*+ 3*ϱ*+ 3−*i*^. Furthermore, the initial edge (*v*_*i*_, *w*_*i*_) of each shortcut has a cost of (1 − *β*)^3*ϱ*+ 2^, while the edges (*w*_*i*_, *s*_*i*_) and (*t*_*i*_, *t*) have complementing cost of (*k* + 1 − *i*)(1 − *β*)^3*ϱ*+ 1^/(*k* + 1) and ${i(1-\beta )^{3\varrho + 1}/(k + 1)+ \sum _{j = 0}^{3\varrho } (1-\beta )^{j}}$. All edges of *H* are free of charge. As a result, the total cost of each shortcut sums up to ${\sum _{j = 0}^{3\varrho + 2} (1-\beta )^{j}}$. Furthermore, the final shortcut edges (*t*_*i*_, *t*) ensure that the agent becomes very expensive to motivate whenever it leaves the main path.

**Fig. 4:**
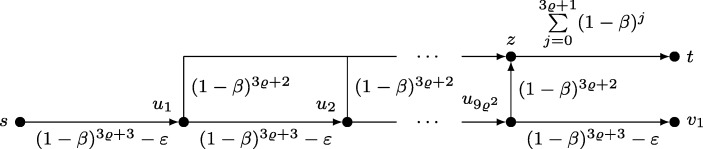
Amplification unit

The amplification unit, which is shown in Fig. 4, consists of an *amplification path* connecting *s* to $u_{9\varrho ^{2}}$ along the intermediate nodes $u_{1}, \ldots , u_{9\varrho ^{2}-1}$. Each edge of the amplification path has a cost of (1 − *β*)^3*ϱ*+ 3^ − *ε*. From every *u*_*i*_ there is also an edge of cost (1 − *β*)^3*ϱ*+ 2^ to a common node *z*. Node *z* is in turn connected to *t* via an edge of cost $\sum _{j = 0}^{3\varrho + 1} (1-\beta )^{j}$. Similar to the embedding unit, the idea of the edge (*z*,*t*) is to ensure that the agent becomes expensive to motivate whenever it leaves the amplification path.

To conclude the proof, we must show that our construction satisfies properties (a) and (b) stated above. We start with (*a*) and assume that *k* node-disjoint paths exist in *H*. Let *G*^′^ be a subgraph of *G* obtained by deleting all edges of *H* that are not part of such a path. Furthermore, we set *r*(*t*) = 1/*β* and *s* = *u*_0_. When located at *u*_*i*_ with 0 ≤ *i* ≤ 9*ϱ*^2^, Lemma 1 suggests that the agent perceives a net cost of − *ε* for traversing (*u*_*i*_, *u*_*i*+ 1_) and then following (*u*_*i*+ 1_, *z*) or the first shortcut of the embedding unit if *i* = 9*ϱ*^2^. Conversely, if *i* > 0, the agent evaluates the net cost of walking along (*u*_*i*_, *z*) to 0. As a result, the agent follows the amplification path until it reaches *v*_1_. From this point on it travels along the main path of the embedding unit until it eventually arrives at *t* for the same reasons given in Theorem 1. This means that *G*^′^ is a motivating subgraph for a reward of *r*(*t*) = 1/*β*.

To prove (b), assume that ${\mathcal I}$ does not have *k* node-disjoint connecting paths and consider any subgraph *G*^′^ of *G*. Furthermore, let the reward *r*(*t*) be at most *ϱ*/*β*. Or goal is to show that *G*^′^ cannot be motivating.

As our first step we argue that the agent certainly abandons the project if it diverts from the amplification path or main path. In case of the amplification path, note that the agent must pass (*z*,*t*) to reach *t* should it divert. However, the cost of (*z*,*t*) is

$$\sum\limits_{j = 0}^{3\varrho+ 1} (1-\beta)^{j} = \sum\limits_{j = 0}^{3\varrho+ 1} \left( 1 - \frac{1}{3\varrho+ 3}\right)^{j} > \sum\limits_{j = 0}^{3\varrho+ 1} \left( 1 - \frac{1}{3\varrho+ 3}\right)^{3\varrho+ 3} > \sum\limits_{j = 0}^{3\varrho+ 1} \frac{1}{3} > \varrho. $$

See Lemma 2 for the second inequality. Clearly, a reward of *r*(*t*) ≤ *ϱ*/*β* is not sufficiently motivating for the agent to traverse this edge. Similarly, if the agent leaves the main path via a shortcut, it must pass an edge (*t*_*i*_, *t*). The cost of these edges is greater than $\sum _{j = 0}^{3\varrho } (1-\beta )^{j} > \varrho $. Again, a reward of *r*(*t*) ≤ *ϱ*/*β* is not motivating enough. Therefore, we may restrict ourselves to subgraphs *G*^′^ in which the amplification path and main path are intact and assume that the agent stays on these paths.

We say that the *i*-th shortcut of *G*^′^ is *degenerate* if the cost of a cheapest path from *v*_*i*_ to *t* via *w*_*i*_ is different from the *target value*$\vartheta = \sum _{j = 0}^{3\varrho + 2} (1-\beta )^{j}$. In particular, a shortcut is degenerate if it does not connect to *t*. Note that by construction every degenerate shortcut must miss the target value by (1 − *β*)^3*ϱ*+ 1^/(*k* + 1) or more. Similar to Theorem 1, the assumption that ${\mathcal I}$ has no solution implies the existence of a degenerate shortcut. By the same argument given in Theorem 1, it is also clear that no degenerate shortcut can cost less than *𝜗* if the agent is to stay on the main path. Without loss of generality we therefore assume the cost of a cheapest path from *v*_*i*_ to *t* via *w*_*i*_ to be greater than *𝜗* for all degenerate shortcuts *i*. We continue to distinguish between two cases depending on whether the first shortcut is degenerate.

If the first shortcut is not degenerate in *G*^′^, then there exists an integer *i* with 1 < *i* ≤ *k* such that the (*i* − 1)-st shortcut is not degenerate, but shortcut *i* is. At *v*_*i*− 1_ the agent’s perceived net cost for taking the current shortcut via *w*_*i*− 1_ is 1 − *βr*(*t*) according to Lemma 1. In contrast, traversing (*v*_*i*− 1_, *v*_*i*_) and taking the next shortcut *i* has a perceived net cost of at least

$$\begin{array}{@{}rcl@{}} &&(1-\beta)^{3\varrho+ 3} -\varepsilon + \beta\left( \sum\limits_{j = 0}^{3\varrho+ 2} (1-\beta)^{j} + \frac{(1-\beta)^{3\varrho+ 1}}{k + 1}\right) - \beta r(t)\\ &=& 1 + \beta\frac{(1-\beta)^{3\varrho+ 1}}{k + 1}-\varepsilon - \beta r(t) > 1 - \beta r(t). \end{array} $$

The inequality holds by choice of *ε*. Moreover, there are no degenerate shortcuts of cost less than *𝜗*. Thus traversing (*v*_*i*− 1_, *v*_*i*_) and walking further along the main path, possibly taking a subsequent shortcut, has a perceived net cost of at least

$$\begin{array}{@{}rcl@{}} &&(1-\beta)^{3\varrho+ 3} -\varepsilon + \beta\left( (1-\beta)^{3\varrho+ 3} -\varepsilon + \sum\limits_{j = 0}^{3\varrho+ 2} (1-\beta)^{j}\right) - \beta r(t)\\ &=& 1+ (1+\beta)\left( \beta\frac{(1-\beta)^{3\varrho+ 3}}{1 + \beta}- \varepsilon\right) - \beta r(t) > 1 - \beta r(t). \end{array} $$

Again, the inequality holds by choice of *ε*. Together, this contradicts the requirement that the agent must not diverge from the main path.

Finally, we consider the case that the first shortcut in *G*^′^ is degenerate with cost greater than *𝜗*. Let *i* be the highest index of a node on the amplification path such that *u*_*i*_ is connected to *t* via (*u*_*i*_, *z*) and (*z*,*t*) in *G*^′^. The perceived net cost of such a path is 1 − *βr*(*t*). Conversely, any path along (*u*_*i*_, *u*_*i*+ 1_), or $(u_{9\varrho ^{2}},v_{1})$ if *i* = 9*ϱ*^2^, has a perceived net cost greater than 1 − *βr*(*t*) as calculated in the last paragraph. Thus the agent leaves the amplification path and abandons. However, if no *u*_*i*_ is connected to *t* via (*u*_*i*_, *z*) and (*z*,*t*), then the lowest perceived net cost at *s* is lower bounded by

$$\begin{array}{@{}rcl@{}} &&(1-\beta)^{3\varrho+ 3}-\varepsilon +\beta\left( 9\varrho^{2}\left( (1-\beta)^{3\varrho+ 3}-\varepsilon\right)+\sum\limits_{j = 0}^{3\varrho+ 2} (1-\beta)^{j}\right) - \beta r(t)\\ &=& 1 - \varepsilon + 9\beta\varrho^{2}\left( (1-\beta)^{3\varrho+ 3}-\varepsilon\right) - \beta r(t). \end{array} $$

Taking into account that *β* = 1/(3*ϱ* + 3) we can further simplify this term to

$$\begin{array}{@{}rcl@{}} &&1 - \varepsilon + 9\varrho^{2}\frac{1/3 + ((1-\beta)^{3\varrho+ 3} - 1/3 -\varepsilon)}{3\varrho+ 3} - \beta r(t)\\ &>& 1 - \varepsilon + 9\varrho^{2}\frac{1/3}{3\varrho+ 3} - \beta r(t) = \varrho + \left( \frac{1}{1+\varrho} - \varepsilon \right) - \beta r(t) > \varrho - \beta r(t). \end{array} $$

Once more, the two inequalities hold by choice of *ε*. Consequently, no *G*^′^ is motivating for a reward of *ϱ*/*β* or less, which proves (b).

To conclude the proof, we must determine a suitable *ϱ*. For this purpose we set *ϱ* = *m*, where *m* is the number of nodes in *H*. As a result the total number of nodes in *G* is *n* = 2 + (9*m*^2^ + 1) + (*m* + 2*k* + 3*m* + 3). The first term accounts for *s* and *t*, the next one for the number of nodes in the amplification unit and the last one for the number of nodes in the embedding unit. Thus we have presented a polynomial reduction. Furthermore, for every positive value *δ* and sufficiently large *k*-DCP instance $\mathcal {I}$ that satisfies $m \geq (\sqrt {6 \delta + 9}+ 3)/\delta $ it holds true that *n* ≤ 9*m*^2^ + 6*m* + 6 ≤ (9 + *δ*)*m*^2^. It follows that $\varrho \geq \sqrt {n/(9 + \delta )}$ and therefore MS-OPT is NP-hard to approximated within any ratio less than $\sqrt {n}/3$.

## Motivation Through Intermediate Rewards

In this section, we study the complexity of motivating agents through the strategic placement of rewards. In this scenario, the task graph must not be pruned. The goal is to minimize the total value of the rewards along the agent’s walk from *s* to *t*. Similar to the previous setting of Sections 3 and 4, a motivating reward configuration within a given budget *b* can be computed in polynomial-time if *β* = 0 or *β* = 1.

### **Proposition 5**

*A motivating reward configuration within budgetbcan be computed in polynomial-time forβ* = 0 *orβ* = 1*.*

### Proof

First, suppose that *β* = 0. In this case, the agent does not care for any future rewards and only traverses edges of cost 0. Let *V*^′^ be the set of nodes that can be reached from *s* for cost 0. Note that *V*^′^ contains exactly those nodes that might be visited by the agent independent of the specific reward configuration. As a result, *G* has a motivating reward configuration if and only if *t* can be reached from every node of *V*^′^ for a cost of 0. Because no rewards need to be placed in this scenario, the budget constraint is always satisfied. Next, assume that *β* = 1. In this case the agent is time-consistent. Let *c* be the cost of a cheapest path from *s* to *t*. Setting *r*(*t*) = *c* yields a motivating and also optimal reward configuration. The required budget is *c*. Clearly both cases, *β* = 0 and *β* = 1 can be solved in polynomial time.

As before, the problem becomes much harder for general *β* ∈ (0, 1). In particular, the corresponding decision problem MOTIVATING REWARD CONFIGURATION (MRC), which we define below, is NP-hard.

### **Definition 4** (MRC)

Given a task graph *G*, a budget *b* and a bias factor *β* ∈ [0, 1], decide the existence of a motivating reward configuration *r* such that the total reward collected on any walk of the agent is at most *b*.

The following proposition establishes membership of MRC in NP.

### **Proposition 6**

*For any task graphG,reward configurationrand bias factorβ* ∈ [0, 1]*,it is possible to decide in polynomial-time ifris motivating within a given budgetb.*

### Proof

The problem is similar to that of Proposition 2. The only difference is that we need to keep track of the budget. For this purpose we modify the algorithm of Proposition 2 in the following way: A cost of *r*(*w*) is assigned to each edge (*v*,*w*) of *G*^′^. Let *c* be the maximum cost among all paths from *s* to *t* in *G*^′^. The budget constraint is satisfied if *c* + *r*(*s*) ≤ *b*. Because *G*^′^ is acyclic, *c* can be computed in polynomial-time.

To show NP-hardness of MRC, we use a reduction from SET PACKING (SP). For convenience, the definition of SP is stated below [5].

### **Definition 5** (SP)

Given a collection of finite sets *S*_1_,…,*S*_*ℓ*_ and an integer *k* ≤ *ℓ*, decide if at least *k* of these sets are mutually disjoint.

We are now ready to prove NP-completeness of MRC. Note that the problem remains hard even if the budget is 0.

### **Theorem 4**

*MRC is NP-complete for any bias factorβ* ∈ (0, 1)*,even ifb* = 0*.*

### Proof

By Proposition 6, we can use any motivating reward configuration *r* within budget *b* as certificate for a “yes”-instance of MRC. This establishes membership of MRC in NP. To prove NP-hardness we present a polynomial-time reduction from SP to MRC. We focus on the case that *b* = 0. A modified reduction for budgets *b* > 0 can be found in the appendix.

**Fig. 5:**
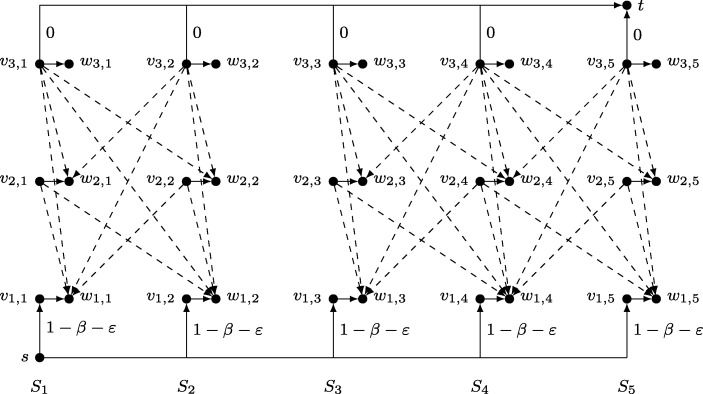
Reduction from the SP instance: *S*_1_ = {*a*}, *S*_2_ = {*a*,*c*}, *S*_3_ = {*b*,*d*}, *S*_4_ = {*d*,*e*}, *S*_5_ = {*e*} and *k* = 3

Let ${\mathcal I}$ be an instance of SP consisting of finite sets *S*_1_,…,*S*_*ℓ*_ and an integer *k* ≤ *ℓ*. We start by constructing an MRC instance ${\mathcal J}$ that has a motivating reward configuration within a budget of *b* = 0 if and only if ${\mathcal I}$ has a solution. Figure 5 depicts the task graph *G* for a small sample instance of SP. In general, *G* consists of a source *s*, a target *t* and 1 ≤ *i* ≤ *k* levels of nodes *v*_*i*,*j*_ with 1 ≤ *j* ≤ *ℓ*. For every *v*_*i*,*j*_ with *i* < *k* there is a so called *upward edge* to every node $v_{i + 1,j^{\prime }}$ on the next level. To maintain readability, upward edges are omitted in Fig. 5. In addition to the upward edges, there is an edge from *s* to every node *v*_1,*j*_ on the bottom level and an edge towards *t* from every node *v*_*k*,*j*_ on the top level. The idea behind this construction is that the agent walks along the upward edges from *s* to *t* in such a way that the nodes $v_{1,j},\ldots ,v_{k,j^{\prime }}$ on its path correspond to a collection of *k* mutually disjoint sets $S_{j},\ldots ,S_{j^{\prime }}$. The cost of the initial edges (*s*,*v*_1,*j*_) and all upward edges $(v_{i,j},v_{i + 1,j^{\prime }})$ is 1 − *β* − *ε*. Note that *β* ∈ (0, 1) might be an arbitrary value with an encoding length that is polynomial in that of ${\mathcal I}$. Moreover *ε* is a positive value satisfying

$$\varepsilon < \min\left\{\frac{(1-\beta)^{2}}{k}, \frac{\beta-\beta^{2}}{k-1+\beta}\right\}. $$

The cost of the edges (*v*_*k*,*j*_, *t*) is 0. To motivate the agent, we add *shortcuts* to *G* that connect every *v*_*i*,*j*_ to *t* via an intermediate node *w*_*i*,*j*_. The first edge (*v*_*i*,*j*_, *w*_*i*,*j*_) has cost 1 and the second edge (*w*_*i*,*j*_, *t*) has cost 0. In Fig. 5 the second edges are omitted for the sake of readability. Note that a reward of value less than 1/*β* can be placed on *w*_*i*,*j*_ without the agent claiming it. Furthermore, if the reward is at least (1 − *ε*)/*β*, all edges $(v_{i-1,j^{\prime }}, v_{i,j})$, or (*s*,*v*_*i*,*j*_) if *i* = 1, become motivating.

We finish our construction by connecting each node *v*_*i*,*j*_ with all nodes $w_{i^{\prime },j^{\prime }}$ for which *i*^′^ < *i* and $S_{j}\cap S_{j^{\prime }}\neq \emptyset $ via a *downward path*. Each downward path consists of two edges: the first one is of cost 0 and the second one is of cost (1 − *β* − *kε*)/(*β* − *β*^2^). In Fig. 5, downward paths are drawn as single dashed edges. The idea behind these paths is to enforce the disjointness constraint of ${\mathcal I}$. In the next paragraph we address this in more detail. But first note that *G* is an acyclic graph that is polynomial in the size of ${\mathcal I}$. It remains to show that ${\mathcal J}$ has a solution if and only if ${\mathcal I}$ has one.

(⇒) First, assume that ${\mathcal I}$ has a solution, i.e. there exists a collection of *k* mutually disjoint sets among *S*_1_,…,*S*_*ℓ*_. To construct a motivating reward configuration *r*, we fix such collection and assign each of its sets *S*_*j*_ to a distinct node level *i*. Furthermore, we set *r*(*w*_*i*,*j*_) = (1 − *ε*)/*β*. The corresponding shortcut from *v*_*i*,*j*_ to *t* is referred to as *active*. By analyzing the agent’s walk, we can show that it visits exactly those nodes *v*_*i*,*j*_ which belong to an active shortcut.

Suppose that the agent is located at *v*_*i*,*j*_ with *i* < *k*. Furthermore, assume that *v*_*i*,*j*_ is the initial node of an active shortcut. There are three options. First, the agent could follow the shortcut to *w*_*i*,*j*_. However, the perceived net cost along this path is *ε* and therefore not motivating. Secondly, the agent could take a downward path. By construction, none of these paths leads to an active shortcut. This means that the agent cannot collect rewards but encounters positive cost, which is not motivating either. The only remaining option is to take one of the upward edges. According to Lemma 1, the agent’s perceived net cost for following the active shortcut onto level *i* + 1 is 0. This is a motivating choice. Conversely, assume that the agent plans a path *P* to *t* that visits a node $v_{i + 1,j^{\prime }}$ such that the corresponding shortcut is not active. We distinguish between four scenarios, none of which is motivating. If *P* includes a downward path, then at most one reward can be located on *P*. In this case the agent perceives a net cost that is at least

$$1-\beta -\varepsilon + \beta\left( \frac{1-\beta-k\varepsilon}{\beta-\beta^{2}} - \frac{1-\varepsilon}{\beta}\right) = \frac{k}{1-\beta}\left( \frac{(1-\beta)^{2}}{k} - \varepsilon\right) >0. $$

The inequality holds by choice of *ε*. This scenario is not motivating. If *P* includes the shortcut at $v_{i + 1,j^{\prime }}$, then *P* contains edges of positive cost but no rewards. Again, this is not motivating. If *P* includes a shortcut on some level *j* > *i* + 1, then the agent must traverse at least two upward edges, but can collect at most one reward. As a result, the perceived net cost is at least

$$1-\beta -\varepsilon + \beta \left( (1-\beta -\varepsilon) + 1 - \frac{1-\varepsilon}{\beta}\right) = \beta(1-\beta-\varepsilon) > \beta\left( \frac{(1-\beta)^{2}}{k}-\varepsilon\right) >0. $$

As always, the last inequality holds by choice of *ε*. Finally, *P* may neither include a downward path nor a shortcut. However, this means that *P* contains edges of positive cost but no rewards. All in all, the only motivating option is to take the upward edge leading to the active shortcut of level *i* + 1.

The same arguments also apply if the agent is located at *s* or on the top level. At *s*, the agent’s only option is to take an upward edge. Therefore, it moves towards the active shortcut of the bottom level. At the top level the agent takes the direct edge to *t*. This incurs no cost. All other options, namely taking a downward path or the current shortcut, are not motivating. We conclude that the agent walks from *s* to *t* along the initial nodes of the active shortcuts. By doing so, the agent cannot collect any rewards. Consequently, *r* is a motivating reward configuration for a budget of *b* = 0.

(⇐) Next, assume that ${\mathcal J}$ has a solution, i.e. there exists a motivating reward configuration *r* such that the agent does not claim any reward. Considering an arbitrary walk of the agent, our goal is to show that the nodes *v*_*i*,*j*_ it visits correspond to *k* disjoint sets *S*_*j*_. A crucial observation is that none of the agent’s walks may include a shortcut or a downward path. This is because a positive reward is needed to lure the agent onto such a path. However, the agent cannot leave shortcuts or downward paths once entered. Therefore, the agent must either claim the reward or abandon. Both scenarios contradict the assumption that *r* is motivating for *b* = 0. We conclude that the agent visits exactly one node *v*_*i*,*j*_ at each level *i*. We call every *v*_*i*,*j*_ that is contained in one of the agent’s walks *active*. Note that there might be more than one active node per level.

In the following we prove that the agent’s lowest perceived net cost is at least (1 − *k*)*ε* at every active node. More precisely, we use backwards induction from level *k* down to level 1 to show that every path from some active node *v*_*i*,*j*_ to *t* has a minimum perceived net cost of (*i* − *k*)*ε*. Additionally, we observe that the only motivating paths along an upward edge $(v_{i,j}, v_{i + 1,j^{\prime }})$ must follow the shortcut at $v_{i + 1,j^{\prime }}$. For the basis of the induction, assume that the agent is at an active node *v*_*k*,*j*_ on the top level. As argued above, the agent cannot take the shortcut or downward path to *t*. Moreover, there are no upward edges. However, the single edge (*v*_*k*,*j*_, *t*) is a motivating path as the agent’s perceived net cost is 0 = (*k* − *k*)*ε*. This proves the basis of the induction.

For the inductive step let *i* < *k* and assume that the agent is located at some active node *v*_*i*,*j*_. Let $v_{i + 1,j^{\prime }}$ be the active node that the agent visits next. Because the agent moves from *v*_*i*,*j*_ to $v_{i + 1,j^{\prime }}$, there must exist a motivating path *P* from *v*_*i*,*j*_ to *t* via $(v_{i,j},v_{i + 1,j^{\prime }})$ that minimizes the perceived net cost. We distinguish four scenarios. First, assume that *i* = *k* − 1 and *P* contains $(v_{k,j^{\prime }}, t)$. This means that the agent anticipates no reward, but positive cost. Clearly, this is impossible as *P* is not motivating. Secondly, assume *P* contains a forward edge $(v_{i + 1,j^{\prime }},v_{i + 2,j^{\prime \prime }})$ and consider the perceived net cost of the remaining part of *P* when viewed from $v_{i + 1,j^{\prime }}$. According to the induction hypothesis, this cost is at least ((*i* + 1) − *k*)*ε*. Furthermore, no reward must be placed at $v_{i + 1,j^{\prime }}$ as this would violate the budget. This means that the perceived net cost of *P* at *v*_*i*,*j*_ increases by *β*(1 − *β* − *ε*) compared to the perceived net cost of *P* at $v_{i + 1,j^{\prime }}$. We conclude that the perceived net cost of *P* at *v*_*i*,*j*_ is at least

$$\left( (i + 1)-k\right)\varepsilon + \beta (1-\beta -\varepsilon) = \left( k-(i + 1)+\beta\right)\left( \frac{\beta-\beta^{2}}{k-(i + 1)+\beta}-\varepsilon\right)>0. $$

The inequality holds by choice of *ε*. Again, *P* is not motivating. Thirdly, assume that *P* contains a downward path out of $v_{i + 1,j^{\prime }}$. In this case, the perceived net cost of *P* at *v*_*i*,*j*_ increases by 1 − *β* − *ε* compared to the perceived net cost of *P* at $v_{i + 1,j^{\prime }}$. This is even more than in the second case. Certainly, *P* cannot be motivating. Finally, assume that *P* contains the shortcut of $v_{i + 1,j^{\prime }}$. When viewed from *v*_*i*,*j*_ instead of $v_{i + 1,j^{\prime }}$, the perceived net cost of *P* increases by 1 − *β* − *ε* and decreases by 1 − *β*. Consequently, the perceived net cost is at least ((*i* + 1) − *k*)*ε* − *ε* = (*i* − *k*)*ε*. Note that this is the only motivating scenario, which concludes the induction step. A similar argument shows that the only motivating paths out of *s* traverse the shortcut of an active node on the bottom level.

The last three paragraphs imply that for every active node *v*_*i*,*j*_ the reward *r*(*w*_*i*,*j*_) must be at least (1 − *ε*)/*β*. Otherwise the agent would not be motivated to move to *v*_*i*,*j*_ when residing at an active node on the previous level *i* − 1, or at *s* if *i* = 1. However, this means that there can be no downward path between two active nodes because the perceived net cost for following the downward path would be at most

$$\beta\left( \frac{1-\beta-k\varepsilon}{\beta-\beta^{2}} - \frac{1-\varepsilon}{\beta}\right) = \frac{(1-k-\beta)\varepsilon}{1-\beta} < (1-k)\varepsilon. $$

This violates the bound established earlier. By construction of *G*, the active nodes *v*_*i*,*j*_ along any of the agent’s walks must correspond to *k* disjoint sets *S*_*j*_. We conclude that ${\mathcal I}$ has a solution.

Finally, we look at the optimization variant of MRC called MRC-OPT.

### **Definition 6** (MRC-OPT)

Given a task graph *G* and a bias factor *β* ∈ (0, 1), determine the infimum of all budgets *b* for which there exists a reward configuration *r* such that the total reward collected on any of the agent’s walks is at most *b*.

The fact that MRC is NP-complete for *b* = 0 immediately implies that MRC-OPT does not permit any efficient approximation algorithm unless P = NP.

### **Corollary 1**

*MRC-OPT is NP-hard to approximate within any ratio greater or equal to* *1.*

## Figures and Tables

**Fig. 6:**
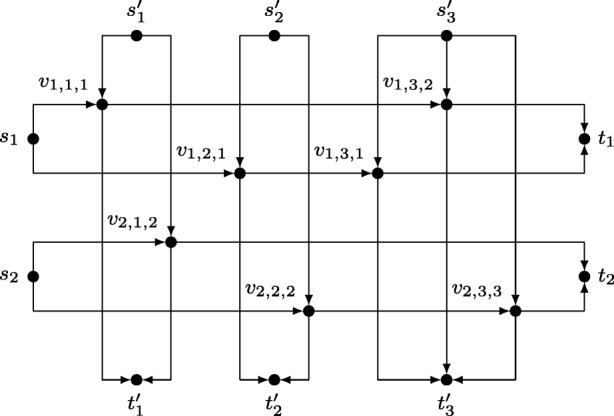
Reduction from the 3-SAT instance: $(\bar {x}_{1}\vee \bar {x}_{2})\wedge (x_{1}\vee x_{2})\wedge (x_{1}\vee \bar {x}_{1}\vee x_{2})$

## Appendix

### **Proposition 7**

*k* *-DCP* *is NP-complete in directed acyclic graphs.*

### Proof

Membership of *k*-DCP in NP is easy to see. To establish NP-hardness we modify Lynch’s reduction to map instances of 3-SAT to instances of *k*-DCP in directed acyclic graphs [11]. Let $\mathcal {I}$ be a 3-SAT instance with *m* clauses *c*_1_…*c*_*m*_ over *n* variables *x*_1_…*x*_*n*_. Furthermore, let $\mathcal {J}$ be an (*m* + *n*)-DCP instance on a directed acyclic graph *H* that is constructed in the following way:

For every variable *x*_*i*_, the terminals *s*_*i*_ and *t*_*i*_ are connected via two node-disjoint paths. One path, the so called high path, corresponds to the case that *x*_*i*_ is set to true. The other path, which we call low path, corresponds to the case that *x*_*i*_ is false. Similar to the variables, there are terminals $s^{\prime }_{j}$ and $t^{\prime }_{j}$ for each clause *c*_*j*_. These terminals are connected via one node-disjoints path for each literal in *c*_*j*_. If the *k*-th literal of *c*_*j*_ is equal to *x*_*i*_, a node *v*_*i*,*j*,*k*_ is added as an intersection between the low path of *x*_*i*_ and the path of the literal. Should the *k*-th literal be negated, i.e. HCode $\bar {x}_{i}$, we add *v*_*i*,*j*,*k*_ to the high path of *x*_*i*_ and the path of the literal instead.

Observe that the incident edges of terminal nodes are either all in-edges or all out-edges. Consequently, terminal nodes cannot be part of a cycle. Furthermore, every other node *v*_*i*,*j*,*k*_ has exactly two out-edges. The first one leads directly to $t_{j}^{\prime }$. The second one follows the high or low path of *x*_*i*_, which eventually ends in *t*_*i*_. Therefore, non-terminal nodes cannot be part of a cycle either. We conclude that *H* is acyclic. Moreover, the size of *H* is polynomial in the encoding length of $\mathcal {I}$. Figure 6 shows an example of *H* for a small sample instance.

Next, we prove that $\mathcal {J}$ is feasible if and only if $\mathcal {I}$ is. (⇒) Given a variable assignment that satisfies $\mathcal {I}$, it is easy to construct *m* + *n* node-disjoint connecting paths in $\mathcal {J}$. For the terminals *s*_*i*_ and *t*_*i*_ choose the high path if *x*_*i*_ is true or the low path if *x*_*i*_ is false. For $s^{\prime }_{j}$ and $t^{\prime }_{j}$ the path of any literal in *c*_*j*_ that evaluates to true can be selected. Because the variable assignment is satisfying, at least one such path must exist. By construction of *H*, all paths are node-disjoint.

(⇐) Recall that every node *v*_*i*,*j*,*k*_ has exactly two out-edges: one that leads to $t_{j}^{\prime }$ and one along the high or low path of *x*_*i*_. As a result, the terminals *s*_*i*_ and *t*_*i*_ can only be connected by a high path or a low path in any solution of $\mathcal {J}$. By construction of *H*, the chosen high and low paths of such a solution directly translate to a variable assignment that satisfies $\mathcal {I}$.

### *Proof of Lemma 1*

If *β* = 0, the claim is easy to verify. Furthermore, for *β* > 0 the geometric series $\sum _{i = 0}^{k-1}(1 - \beta )^{i}$ evaluates to (1 − (1 − *β*)^*k*^)/*β*, which in turn implies that

$$(1 - \beta)^{k} + \beta\left( \sum\limits_{i = 0}^{k-1}(1 - \beta)^{i}\right) = (1-\beta)^{k} + \beta\left( \frac{1-(1-k)^{k}}{\beta}\right) = 1. $$

### *Proof of Lemma 2*

Recall that the sequence (1 − 1/*n*)^*n*^ converges towards 1/*e*. In particular, the function (1 − 1/*n*)^*n*^ is monotonically increasing for *n* ≥ 1. As a result, it holds true that

$$\left( 1-\frac{1}{3\varrho+ 3}\right)^{3\varrho+ 3} \geq (1-{1/6})^{6} > {1/3}. $$

### *Proof of Theorem 4 for arbitrary**b*

In the following we sketch how the reduction in the proof of Theorem 4 can be generalized to prove NP-hardness for arbitrary budgets *b* > 0. We assume that encoding length of *b* is polynomial in that of ${\mathcal I}$. To establish NP-hardness we modify the previous task graph in the following way. We rename the target node to *t*^′^ and insert a new target node *t*. Both nodes are connected via an edge (*t*^′^, *t*) of cost *β**b*. As a result, a reward of *b* or more must be placed at *t* for the agent to traverse (*t*^′^, *t*) and finish. However, when the agent reaches *t* it has certainly collect this reward. This means that every motivating reward configuration *r* within the budget constraints must set *r*(*t*) = *b*. Furthermore, no other rewards must be collected by the agent.

To compensate for the extra reward at *t*, some of the edges in *G* become more expensive. The price of each edge (*v*_*k*,*j*_, *t*^′^) as well as the initial edge of every shortcut increases by an additional (1 − *β*)*β**b*. Their new cost is (1 − *β*)*β**b* and 1 + (1 − *β*)*β**b*. The price of upward edges is increased by (1 − *β*)^2^*β**b* to (1 − *β* − *ε*) + (1 − *β*)^2^*β**b*. Finally, the price of the second edge of each downward path is increased by (1 − *β*)*b* to (1 − *β* − *k**ε*)/(*β* − *β*^2^) + (1 − *β*)*b*. All other edges have the same cost as before.

Clearly, the size of $\mathcal {J}$ is polynomial in that of ${\mathcal I}$. Moreover, the same reasoning that we used in the special case of *b* = 0 shows that ${\mathcal I}$ has a solution if and only if ${\mathcal J}$ has one. To verify this claim it is helpful to observe how the reward at *t* and the additional edge costs cancel each other out according to Lemma 1.
